# Biocontrol and plant growth promotion potential of endophytic *Bacillus subtilis* JY-7-2L on *Aconitum carmichaelii* Debx.

**DOI:** 10.3389/fmicb.2022.1059549

**Published:** 2023-01-10

**Authors:** Lan Zou, Qian Wang, Rongxing Wu, Yaopeng Zhang, Qingshan Wu, Muyi Li, Kunhao Ye, Wei Dai, Jing Huang

**Affiliations:** ^1^School of Life Science and Engineering, Southwest University of Science and Technology, Mianyang, China; ^2^Mianyang Academy of Agricultural Science, Mianyang, China

**Keywords:** endophyte, *Bacillus subtilis*, biocontrol, southern blight, field trials, *Aconitum carmichaelii* Debx.

## Abstract

*Aconitum carmichaelii* Debx. is a famous medicinal plant rich in alkaloids and widely used to treat various human diseases in Asian countries. However, southern blight caused by *Sclerotium rolfsii* severely hampered the yield of *A. carmichaelii*. Beneficial microbe-based biological control is becoming a promising alternative and an environmentally friendly approach for the management of plant diseases. In this study, we evaluated the biocontrol potential of an endophytic bacterial strain JY-7-2L, which was isolated from the leaves of *A. carmichaelii*, against southern blight *in vitro* and by a series of field experiments. JY-7-2L was identified as *Bacillus subtilis* based on multi-locus sequence analysis. JY-7-2L showed strong antagonistic activity against *S. rolfsii in vitro* and on *A. carmichaelii* root slices by dual-culture assay. Cell-free culture filtrate of JY-7-2L significantly inhibited the hyphal growth, sclerotia formation, and germination of *S. rolfsii*. In addition, volatile compounds produced by JY-7-2L completely and directly inhibited the growth of *S. rolfsii*. Furthermore, JY-7-2L was proved to produce hydrolytic enzymes including glucanase, cellulase, protease, indole acetic acid, and siderophore. The presence of *bacA*, *fenA*, *fenB*, *fenD*, *srfAA*, and *baeA* genes by PCR amplification indicated that JY-7-2L was able to produce antifungal lipopeptides and polyketides. Field trials indicated that application of the JY-7-2L fermentation culture significantly reduced southern blight disease severity by up to 30% with a long-acting duration of up to 62 days. Meanwhile, JY-7-2L significantly promoted the fresh and dry weights of the stem, main root, and lateral roots of *A. carmichaelii* compared to non-inoculation and/or commercial *B. subtilis* product treatments. Taken together, JY-7-2L can be used as a promising biocontrol agent for the control of southern blight in *A. carmichaelii*.

## 1. Introduction

*Aconitum carmichaelii* Debx. is a famous herbaceous perennial flowering medicinal plant used widely in China, Korea, Japan, India, and other Asian countries (Miao et al., 2019). The most frequently used herbal drug derived from *A. carmichaelii* is the processed lateral (daughter) root called “Fuzi,” which is an essential ingredient in many traditional Chinese medicine prescriptions (Chan et al., 2021). The major active substances of Fuzi are alkaloids with a wide range of biological and pharmacological effects (Zhou et al., 2015; Wu et al., 2018; Miao et al., 2019). Fuzi has been widely used to treat various diseases, for example, rheumatoid arthritis, cardiovascular diseases, tumors, and depression, for 2,000 years in Chinese clinics (Zhou et al., 2015). During the pandemic of COVID-19, Fuzi has been suggested as one of the key traditional Chinese medicinal herbs for the treatment of critical patients by the National Health Commission of China.^1^ *A. carmichaelii* plants are mainly cultivated in Sichuan, Yunnan, and Shaanxi provinces in China (Yu et al., 2016). Jiangyou in Sichuan province has been considered the geo-authentic area of *A. carmichaelii* for over 1,300 years because Fuzi in this place has the highest quality (Yu et al., 2016). Apart from being widely used in China, *A. carmichaelii* products from the geo-authentic area are also widely exported to European countries. The needs for Fuzi are huge and increase year by year.

However, *A. carmichaelii* has faced great challenges from soil-borne diseases such as southern blight caused by *Sclerotium rolfsii* and root rot caused by *Fusarium oxysporum*. Southern blight has led to a 60% reduction in the productivity of *A. carmichaelii* in severe fields each year (Li et al., 2019). Specifically, in the geo-authentic area, the redundant daughter roots will be removed to keep only two or three per plant during the growth period of *A. carmichaelii*, which further aggravates southern blight occurrences (Dai et al., 2016). *S. rolfsii* usually infects the intermediate part of the root and stem, causing wilt, rot, and death in *A. carmichaelii* plants (Li et al., 2022). Noteworthy, *S. rolfsii* can form a special structure, i.e., the sclerotium, which is generated from the aggregation of vegetative hyphae enclosed by a brown, tough surface (Li et al., 2019). Sclerotia can survive for a long time (several years) in soil, even under harsh conditions, and they are resistant to fungicides (Li et al., 2017; Jacob et al., 2018). Currently, chemical fungicides are often applied to control southern blight in the cultivation areas of *A. carmichaelii* but with little efficacy and large environmental pollution drawbacks (Fira et al., 2018). The development of safe and eco-friendly biological control approaches is urgently needed for the sustainable development of *A. carmichaelii* industry (Zhou et al., 2022).

Beneficial microbe-based biological control has been demonstrated to be a promising alternative for soil-borne disease control. The screening of highly effective microbial agents is a preliminary step to successful biological control. Endophytes that colonize for the whole or part of their lifecycle and co-evolve with the host plants have attracted great attention for their plant growth promotion and antagonistic activity toward pathogens and are thus important resources for bio-fertilizers or biocontrol agents (De Silva et al., 2019). Among endophytes identified, the genus *Bacillus* has been proven to be one of the dominant genera in many plants and exhibits plant growth promotion and biocontrol properties. For example, *B. tequilensis* YYC155 represented an effective biocontrol agent against anthracnose disease in *Camellia oleifera* (Zhou et al., 2022). *Bacillus velezensis* SDTB038 exhibited promising biocontrol potential for the *Fusarium* crown and root rot of tomato (Chen et al., 2022). *Bacillus stratosphericus* was demonstrated to promote ginseng plant growth and inhibit the growth of root rot fungal pathogens (Durairaj et al., 2018). *Bacillus subtilis* MBI600 was highlighted as a biocontrol agent by promoting plant growth and antagonizing against different pathogens of cucumber (Samaras et al., 2020). The modes of action of *Bacillus* spp. in plant growth promotion and biocontrol of pathogens involve direct and indirect mechanisms. Nitrogen fixation, siderophore and phytohormone production, and phosphorus and potassium solubilization are usually the direct mechanisms used by *Bacillus* for plant growth promotion (Backer et al., 2018; Blake et al., 2021). While the production of secondary metabolites, including hydrolytic enzymes, antibiotics, and volatile organic compounds, is usually the direct mode of action by *Bacillus* to inhibit pathogens (Xu et al., 2019; Niu et al., 2020). In addition, the induction of systemic resistance is usually the indirect mechanism employed by *Bacillus* for plant protection against pathogens (Shafi et al., 2017; Wu et al., 2019; Niu et al., 2020).

Although many studies uncovered the potential of beneficial *Bacillus* strains on plant growth promotion and biocontrol of diseases under controlled conditions either in the laboratory or greenhouse, examples of successful application of microbial biocontrol agents in commercial field-based crop production are rare (Mazzola and Freilich, 2017; Niu et al., 2020). Since field conditions are more complex than controlled laboratory or greenhouse, microbial biocontrol agents may not persist or be outcompeted by indigenous microbes. For example, Stadler and von Tiedemann (2014) reported that *Microsphaeropsis ochracea* showed promising potential to inhibit the growth of the soil-borne fungal pathogen *Verticillium longisporum in vitro* and *in vivo* under laboratory conditions but exhibited no significant antagonistic effectiveness under field conditions. Therefore, it is recommended to evaluate biocontrol activity initially *in vitro* and *in vivo* under laboratory conditions, followed by field trials to check the adaptability and persistence (effective duration) of biocontrol agents (De Silva et al., 2019). However, reports on the evaluation of the biocontrol efficacy of microbe-based biocontrol agents under field conditions were rare, particularly on medicinal plants (Eljounaidi et al., 2016; Wang et al., 2021). Li et al. (2019, 2020) reported that *Penicillium griseofulvum* CF3 and *Streptomyces pactum* Acy12 showed biocontrol efficacy on root diseases of *A. carmichaelii*. Whether endophytic *Bacillus* strains from *A. carmichaelii* could be highlighted as a good biocontrol agent for southern blight under field conditions remains unclear. Therefore, the objectives of this study were to (1) investigate the phylogenetic status of an endophytic bacterial strain JY-7-2L isolated from the leaves of *A. carmichaelii*; (2) evaluate the antagonistic activity of JY-7-2L against *S. rolfsii* and potential mode of action *in vitro*; (3) evaluate the biocontrol efficacy of JY-7-2L under field conditions; and (4) assess the plant growth promotion ability of JY-7-2L *in vitro* and under field conditions.

## 2. Materials and methods

### 2.1. Bacterial and fungal strains and their growth conditions

Endophytic bacterial strain JY-7-2L was isolated from healthy *A. carmichaelii* Debx. plants collected from Jiangyou, Sichuan province in China. *A. carmichaelii* plants without any disease symptoms were dug out of the soil without causing any physical wounds and transferred to the laboratory within 24 h in the harvest season (June) of 2020. Then, the plants were washed under tap water to remove soil and dust. Organs of the plants (roots, stems, and leaves) were cut into small pieces or slices and surface sterilized. Briefly, materials were immersed in 75% ethanol for 2 min and washed two times with sterilized water. Then NaClO (2% Cl v:v) was added to immerse the organs for 8 min followed by rinsing with sterilized water 5 times. An aliquot (10 μl) of the last wash was spotted on Luria-Bertani (LB) agar medium to check the efficacy of surface sterilization. There was no colony growth, indicating that the sterilization was successful, and the materials could be used for endophytic bacterial isolation. Sterilized organs were ground into smaller pieces, or homogenate, which were further placed on LB agar medium and incubated at 28°C for 2 weeks. Colonies around the materials were selected for purification. A white, irregularly shaped, non-transparent colony was isolated, and we named it JY-7-2L. This strain was purified three times on LB agar medium, and then a single colony was picked into 10 ml LB liquid medium and incubated at 28°C with shaking at 150 rpm/min until the logarithmic phase (OD_600_ = 1.2). Thereafter, this cell culture was stored in glycerol (final concentration: 25%) at −80°C.

A total of four pathogenic strains were used in the current study. *S. rolfsii*-1 and *S. rolfsii*-2, the causing pathogens for southern blight in *A. carmichaelii*, were provided by Dr. Yulong Li from Northwest A&F University (Li et al., 2019) and KY from Yunnan Agricultural University, respectively. *F. oxysporum*-1 and *F. oxysporum*-2, which were the causing pathogens for root rot in *A. carmichaelii* were provided by KY from Yunnan Agricultural University. The four strains were grown on a PDA medium, and all have been confirmed for pathogenicity by Koch’s rule for *A. carmichaelii*. *S. rolfsii*-1 showed the strongest pathogenicity on *A. carmichaelii* and thus was used as the target pathogen in the cell-free culture filtrate and volatile assays.

Commercial *B. subtilis*-208 product (PD20150091) was bought from Zhongbao crop protection Co., Ltd. (Hubei, China). This commercial *B. subtilis* was stored in a wettable powder that contained more than 100 billion active spores per gram.

### 2.2. Phylogenetic analysis of JY-7-2L

Strain JY-7-2L was grown on LB agar medium until single colonies appeared. A single colony was picked and inoculated into 10 ml LB liquid medium and incubated at 28°C for 3 days with shaking at 150 rpm/min to make cell culture. Then genomic DNA was extracted using the Bacterial Genomic DNA Isolation Kit (B518225, Sango Biotech, Shanghai, China) following the manufacturer’s instructions. The quantity of DNA was checked by electrophoresis in a 0.8% agarose gel. Housekeeping genes including 16S rRNA, *atpD* (involved in the synthesis of ATP synthase beta subunit), *rpoB* (responsible for the synthesis of RNA polymerase subunit beta), and *gyrA* (involved in the synthesis of DNA gyrase subunit A) were PCR amplified following the primers and procedures indicated in Supplementary Table 1. The PCR products were purified using a DNA gel extraction kit (GE0101-200, Tsingke, China) following the manufacturer’s instructions and sent to Tsingke Biotechnology Co., Ltd, China, for sequencing. Sequences were then blasted into the National Center for Biotechnology Information (NCBI) database, and reference strains were collected. Phylogenetic trees based on a single gene and multi-locus sequence analysis (MLSA) of concatenated sequences of *atpD*, *rpoB*, and *gyrA* were constructed using the Neighbor-Joining method by MEGA X with 1,000 bootstrapped replicates. The sequences for 16S rDNA, *atpD*, *rpoB*, and *gyrA* were deposited in the Genbank database under the accession numbers: OP363704 and OP391483–OP391485.

### 2.3. Antifungal activity measurement

Antifungal activity of JY-7-2L was measured *in vitro* and on root slices of *A. carmichaelii* by a dual-culture assay. Four fungal pathogens (*S. rolfsii*-1, *S. rolfsii*-2, *F. oxysporum*-1, and *F. oxysporum*-2) were prepared on PDA agar medium. The PDA agar disc (diameter of 2.0 mm) containing each pathogen was inoculated in the center of a new PDA agar medium. The cell culture of JY-7-2L was prepared as mentioned above. Then four droplets of JY-7-2L cell culture (10 μl, OD_600_ = 0.2) were inoculated on the PDA agar medium 2 cm away from the pathogen in four directions, respectively. Pathogen inoculation alone was used as a control. Then the diameters of the pathogens were measured after 7 days of inoculation at 25°C. The experiments were performed in triplicates. Lateral roots of healthy *A. carmichaelii* were surface sterilized and cut into 1 cm-thick slice. JY-7-2L cell culture (10 μl, OD_600_ = 0.2) was inoculated on the root slice and placed in a petri dish supplemented with a piece of wet filter paper. After drying, a pathogen disc was placed on the slice. Then, Petri dishes were sealed with parafilm and incubated at 25°C for 7 days. Thereafter, the diameters of the pathogens on the slices were recorded. One milliliter of sterilized water was added to the filter paper if needed. Pathogen discs alone were used as a control. The experiments were conducted three times. The inhibition rate was calculated by the following formula:


Inhibitionrate(%)=(Dc-Db)/Dc×100

where Dc is the diameter of pathogens in the control treatment and Db is the diameter of pathogens co-cultured with JY-7-2L.

### 2.4. Effect of cell-free culture filtrate of JY-7-2L on hyphal growth, sclerotia formation, and germination of *S. rolfsii*

JY-7-2L cell culture was prepared in 100 ml LB broth medium as mentioned above. The supernatant was then collected by centrifugation and filtered by a 0.22 μm microporous membrane (Sango Biotech, Shanghai, China) to obtain cell-free culture filtrate. Then the filtrate was added to autoclaved, warm PDA agar medium (1:4 v:v). The mixture was then poured into petri dishes and cooled to make plates. PDA agar plates containing only LB broth medium were used as a control. *S. rolfsii*-1 was used as the target pathogen because this strain showed the strongest pathogenicity on *A. carmichaelii*. *S. rolfsii* was inoculated in PDA agar medium and incubated at 25°C for 7 days until mature sclerotia formed. Sclerotia were then collected. Agar plugs (2.0 mm in diameter) or sclerotia (12 per plate) of *S. rolfsii* were placed in the center of the plates and incubated at 25°C for up to 9 days. The diameters of pathogens, number of sclerotia, and number of germinated sclerotia were recorded. The experiments were performed in triplicates. The sclerotia germination rate was calculated by the following formula:


Germinationrate(%)=Ng/Nt×100

where Ng is the number of germinated sclerotia and Nt is the total number of sclerotia.

### 2.5. Volatile assay

Dual culture by sealing two agar base plates up and down, i.e., one was an LB agar plate inoculated with JY-7-2L and the other was a PDA agar plate with a pathogen disc inoculum, was carried out to test whether JY-7-2L produced antifungal volatile compounds. Noteworthy, *S. rolfsii*-1 was used as the target pathogen in this assay because this strain showed the strongest pathogenicity on *A. carmichaelii*. Empty LB agar medium coupled with pathogen-containing PDA agar medium was used as a control. The plates were incubated at 25°C for 7 days, the diameters of the pathogens were recorded, and the inhibition rate was calculated as mentioned previously. The experiment was performed in triplicates.

### 2.6. Indole acetic acid and siderophore production by JY-7-2L

Indole acetic acid (IAA) production by JY-7-2L was carried out using the Salkowski method. A single colony of JY-7-2L was inoculated into 5 ml liquid LB medium supplemented with 1 ml of L-tryptophan (2.5 mg/ml) and incubated at 28°C for 2 days. The supernatant (2 ml) was collected and added to 4 ml Salkowski reagent [1 ml 0.5 M FeCl_3_ and 49 ml HClO_4_ (35% v:v)], followed by incubation at room temperature under dark conditions for 30 min. An absorbance value of 530 nm was detected by a spectrophotometer (HA1707241-201). Pure IAA (Sigma-Aldrich, United States) was prepared in different concentrations (0, 5, 10, 20, 40, and 60 mg/L) following the same procedure mentioned above to make a standard curve.

Siderophore production by JY-7-2L was performed on chrome azurol-s (CAS) agar medium. A single colony of JY-7-2L was inoculated into a 10 ml LB liquid medium and incubated at 28°C at 150 rpm/min until the logarithmic phase (OD_600_ = 1.0). An aliquot of bacterial culture (10 μl, OD_600_ = 0.2) was spotted on a CAS agar plate for siderophore production (Xu et al., 2019). The appearance of an orange transparent zone indicated positive activity in siderophore production by the strain after 7 days of incubation at 28°C.

### 2.7. Hydrolytic enzymes production by JY-7-2L

Cellulase, glucanase, and protease production by JY-7-2L were assessed by the plate method. The cell culture of JY-7-2L was prepared as mentioned above. An aliquot of bacterial culture (10 μl, OD_600_ = 0.2) was spotted on carboxymethyl cellulose (CMC) agar medium, glucan agar medium, and skim milk agar medium stained by Congo red (0.2%, w:v) to detect cellulase, glucanase, and protease production by JY-7-2L, respectively (Xu et al., 2019; Ben Khedher et al., 2021). The plates were incubated at 28°C for 7 days. The presence of a hydrolytic halo indicated positive activity in enzyme production by JY-7-2L.

### 2.8. Amplification of secondary metabolites synthesis genes

The genomic DNA of JY-7-2L was extracted as mentioned above. Genes involved in fengycin (*fenA*, *fenB*, and *fenD*), surfactin (*srfAA*), bacilysin (*bacA*), and bacillaene (*baeA*) were PCR amplified. The primers, reaction mixture, and corresponding procedures followed those by Ben Khedher et al. (2021). PCR products were purified and sequenced at Tsingke Biotechnology Co., Ltd, China. Sequences were then blasted in the NCBI database, and phylogenetic trees were constructed by the Neighbor-Joining method in MEGA X with 1,000 bootstrap values. The sequences for *fenA*, *fenB*, *fenD*, *srrA*, and *bacA* have been deposited in the Genbank database under the accession numbers: OP541878–OP541881 and OP542204.

### 2.9. Field experiments

The biocontrol and growth promotion potential of JY-7-2L on *A. carmichaelii* was assessed by field experiments in the research field of Southwest University of Science and Technology (SWUST, N 31° 32′ 01″, E 104° 41′ 43″, elevation 470 m a.s.l., 5-year mean annual temperature 17.82°C) in Jiangyou in 2021 and 2022 and Qingchuan, Guangyuan (N 32° 27′ 21″, E 105° 10′ 12″, elevation 1,300 m a.s.l., 5-year mean annual temperature 13.24°C) in 2021. The soil types were paddy soil in Jiangyou and dark brown forest soil in Qingchuan. The cultivar of *A. carmichaelii* was MianFu No. 1, which was bred and provided by the Mianyang Academy of Agricultural Science at both sites. Field experiments followed the randomized block design principle. Each plot (1.0 m × 3.7 m = 3.7 m^2^) contained 50 plants. Each plot consisted of two plant rows, and the row spacing was 30 cm. Each row consisted of 25 plants, and the plant spacing was 14 cm. The preparation of JY-7-2L cell culture and the commercial *B. subtilis* product solution was as follows: A single colony of JY-7-2L was picked into 1L LB broth medium and incubated at 28°C with shaking at 150 rpm/min for 7 days until logarithmic phase. Then the concentration was adjusted to 1 × 10^9^ cells/ml for inoculation (10 ml/plant). The wetted powder of the commercial *B. subtilis* was dissolved in LB broth and adjusted to 1 × 10^9^ spores/ml for inoculation (10 ml/plant). The sterilized LB broth was always used as a blank control (CK or non-inoculation). Plant management adhered strictly to local customs; specifically redundant lateral roots were removed from the SWUST site in May (lateral roots formation stage).

The field experiments in SWUST were conducted as follows: Two treatments were performed in 2021, which included JY-7-2L cell culture and CK, while a commercial *B. subtilis* product was added as the positive control (three treatments in total) in 2022. Each treatment was performed in six replicated blocks (*n* = 6). The cultivation time was in the middle of November, and the harvest time was in the middle of June in the following year. Southern blight usually occurs at the beginning of May when the weather is getting warmer in this area (Dai et al., 2016). Inoculation was performed at the beginning of May on the wounded root parts caused by the removal of redundant lateral roots using sterilized syringes in both years.

The field experiment in Qingchuan contained two treatments, i.e., JY-7-2L cell culture and CK. Each treatment was conducted in four replicate blocks (*n* = 4). The temperature was lower in the Qingchuan area, so the growth period of *A. carmichaelii* was longer. The cultivation time was in early December, and the harvest time was in the middle of November in the following year. It was noteworthy that *A. carmichaelii* roots from Qingchuan were used as mother roots for the Jiangyou area, so the redundant lateral roots would not be removed in this area. The occurrence of southern blight usually happens in August when the weather is warm, which facilitates the growth and infection of *S. rolfsii*. Inoculation was performed in August at the lateral root formation stage, administered directly to the roots by sterilized syringes.

Southern blight disease occurrences were investigated consistently until harvest time. The numbers of plants with southern blight symptoms (wilt, rot, or death of the plant with white hypha or brown sclerotia of *S. rolfsii* appearing on the roots) were recorded. Because southern blight is a root disease, even light symptoms on leaves or stems indicate a severe infection on the roots by the pathogen. The disease occurrences and biocontrol efficacy were calculated by the following equations:


Diseaseoccurrence(%)=Nd/Nt×100

where Nd is the number of diseased plants and Nt is the number of total plants.


Biocontrolefficacy(%)=(DOc-DOt)/DOc×100

where DOc is the disease occurrence under control and DOt is the disease occurrence of bacterial inoculation treatment.

After harvest, five plants in each plot were randomly selected and the fresh and dry weights of the stem, main root, and lateral roots (Fuzi) were measured.

### 2.10. Statistical analysis

Pathogen hyphal growth, sclerotia formation and germination rate, disease occurrence, and plant biomass were analyzed by one-way analysis of variance followed by a least significant difference test. Pairwise comparisons of group means were done by the Student’s *t*-test. Prior to the ANOVA and *t*-test, the normality of the distribution and homogeneity of the variance were checked with Shapiro–Wilk and Levene’s tests, respectively. SPSS v25.0 (SPSS Inc., Chicago, IL, USA) was used to perform all the statistical analyses. Differences were considered significant at *p* < 0.05.

## 3. Results

### 3.1. Phylogenetic characterization of JY-7-2L

**FIGURE 1:**
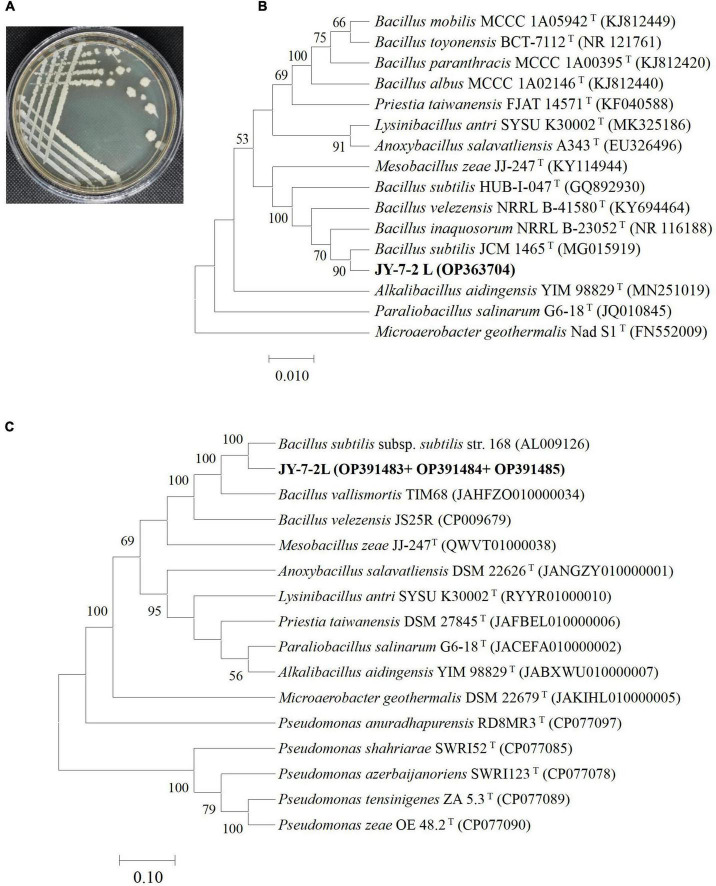
Phylogenetic characterization of JY-7-2L. **(A)** Morphology of JY-7-2L on Luria-Bertani (LB) agar medium; **(B)** phylogenetic tree based on 16S rRNA gene; and **(C)** a concatenated alignment of *atpD*, *gyrA*, and *rpoB* gene sequences. The trees were constructed using the Neighbor-Joining method with 1,000 bootstrap replicates. A bootstrap value greater than 50% was shown on the branches. Accession numbers were indicated in brackets.

JY-7-2L strain displayed off-white, non-transparent, and non-regularly shaped colony morphology, as shown in Figure 1A. A total of 4 housekeeping genes were amplified from the genomic DNA of JY-7-2L, including 16S rRNA, *atpD*, *gyrA*, and *rpoB*, which generated a product fragment of 1,451, 529, 974, and 562 bp, respectively. Phylogenetic trees based on each gene and multi-gene sequences (a concatenated sequence of *atpD*, *gyrA*, and *rpoB*) were constructed using the Neighbor-Joining method. JY-7-2L clustered together with *B. subtilis* JCM 1465*^T^* sharing 100% similarity in the phylogenetic tree based on the 16S rRNA gene sequence (Figure 1B). Single housekeeping gene-based phylogenetic trees showed the same result with MLSA, where JY-7-2L clustered together with *B. subtilis* (97.4% similarity in MLSA) (Figure 1C and Supplementary Figures 1–3). Taken together, JY-7-2L was identified as *B. subtilis*.

### 3.2. Antagonistic activity of JY-7-2L against fungal pathogens

**FIGURE 2:**
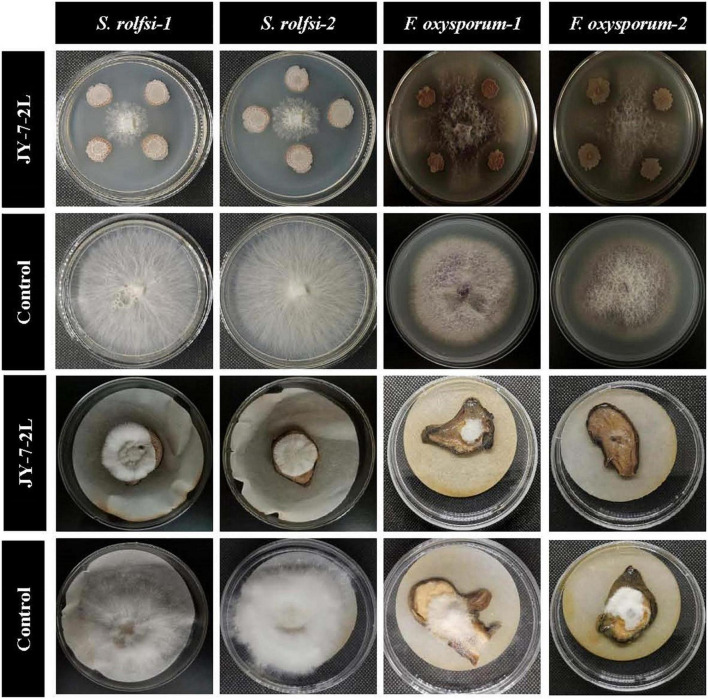
Antagonistic activity of JY-7-2L against *Sclerotium rolfsii* and *Fusarium oxysporum in vitro* and on *Aconitum carmichaelii* root slices by dual culture method.

JY-7-2L could inhibit the growth of four fungal pathogens (Figure 2). The inhibition rate of JY-7-2L against *S. rolfsii*, which induces southern blight, and *F. oxysporum*, which causes root rot on *A. carmichaelii*, ranged from 70.59 to 71.57% and from 67.23 to 68.65% *in vitro* (Table 1), respectively. The antagonistic ability of JY-7-2L against fungal pathogens on *A. carmichaelii* root slices was further investigated. No disease symptom was detected after inoculation of JY-7-2L cell culture on root slices, indicating that JY-7-2L was non-pathogenic to *A. carmichaelii*. The results showed that JY-7-2L prevented the growth of *S. rolfsii* by up to 67.92% and *F. oxysporum* by up to 48.10% on root slices, respectively (Table 1). Therefore, JY-7-2L showed good antagonistic activity against the fungal pathogen *A. carmichaelii*.

**TABLE 1:** Antagonistic activity of JY-7-2L against *Sclerotium rolfsii* and *Fusarium oxysporum in vitro* and on *Aconitum carmichaelii* root slices by dual culture method.

Isolate	Inhibition rate on PDA plates (%)				Inhibition rate on *A. carmichaelii* root slices (%)			
	***S. rolfsii*-1**	***S. rolfsii*-2**	***F. oxysporum-1***	***F. oxysporum-2***	***S. rolfsi*-1**	***S. rolfsi*-2**	***F. oxysporum-1***	***F. oxysporum-2***
JY-7-2L	70.59 ± 1.36	71.57 ± 1.23	67.23 ± 1.41	68.65 ± 2.57	54.04 ± 7.16	67.92 ± 6.03	48.10 ± 5.72	33.00 ± 5.68

Data were presented by mean with a standard error of three replicates.

### 3.3. Effect of the cell-free culture of JY-7-2L on hyphal growth and sclerotia formation of *S. rolfsii*

**FIGURE 3:**
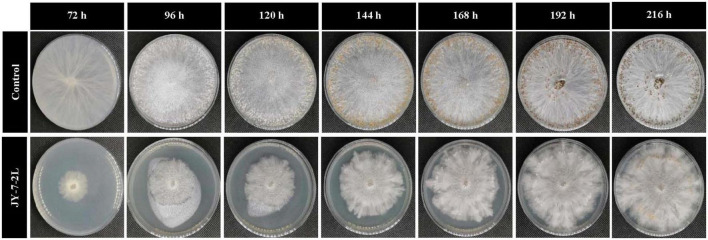
Effect of cell-free culture filtrate of JY-7-2L on hyphal growth and sclerotia formation of *Sclerotium rolfsii.*

Hyphal growth of *S. rolfsii* was inhibited by cell-free culture filtrate of JY-7-2L, and this effect persisted for up to 192 h (Table 2 and Figure 3). Specifically, significant inhibition was detected after 72 h of incubation (inhibition rate: 72.55%). However, the inhibitory ability of cell-free culture decreased over time. *S. rolfsii* started to form sclerotia after 96 h of incubation in the control treatment, while the cell-free culture of JY-7-2L postponed the formation of sclerotia in *S. rolfsii* until 168-h post-incubation. In addition, the number of mature sclerotia formed by *S. rolfsii* in the control treatment was 430.67, while cell-free culture of JY-7-2L significantly reduced the number of sclerotia (48.00) (Table 2 and Figure 3).

**TABLE 2:** Effect of cell-free culture filtrate of strain JY-7-2L on hyphal growth and sclerotia formation of *Sclerotium rolfsii*.

Isolate	Diameter of *S. rolfsii* colony (cm)						No. of sclerotia
	**72 h**	**96 h**	**120 h**	**144 h**	**168 h**	**192 h**	**216 h**
CK	8.50 ± 0.00 a	8.50 ± 0.00	8.50 ± 0.00	8.50 ± 0.00	8.50 ± 0.00	8.50 ± 0.00	430.67 ± 18.61 A
JY-7-2L	2.33 ± 1.22 b	3.23 ± 1.70	4.47 ± 1.67	5.47 ± 1.33	6.17 ± 1.50	6.73 ± 1.32	48.00 ± 25.29 B

Data were presented by means ± SE. Different lower-case letters and upper-case letters indicate significant differences by *t*-test (lower-case letters for *p* < 0.05, upper-case letters for *p* < 0.01).

### 3.4. Effect of cell-free culture filtrate of JY-7-2L on sclerotia germination of *S. rolfsii*

**FIGURE 4:**
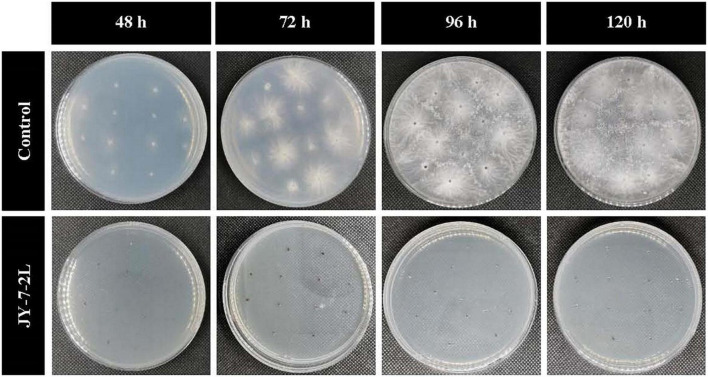
Effect of cell-free culture filtrate of JY-7-2L on sclerotia germination of *Sclerotium rolfsii*.

No bacterial colonies appeared on the PDA plates supplemented with cell-free culture filtrate from JY-7-2L, which indicated no bacterial contamination. Sclerotia germination was completely inhibited by the cell-free culture of JY-7-2L (Table 3 and Figure 4). Sclerotia started to germinate at 48-h post-incubation on PDA plates (CK), while no sclerotia germinated on the cell-free culture of JY-7-2L added PDA plates even after 120-h incubation (Table 3 and Figure 4). Overall, the cell-free culture of JY-7-2L showed a significant inhibitory effect on sclerotia germination of *S. rolfsii* compared to CK.

**TABLE 3:** Effect of JY-7-2L cell-free culture filtrate on sclerotia germination of *Sclerotium rolfsii*.

Isolate	Sclerotia germination rate (%)			
	**48 h**	**72 h**	**96 h**	**120 h**
CK	100.00 ± 0.00 A	100.00 ± 0.00 A	100.00 ± 0.00 A	100.00 ± 0.00 A
JY-7-2L	0.00 ± 0.00 B	0.00 ± 0.00 B	0.00 ± 0.00 B	0.00 ± 0.00 B

Data were represented by the means ± SE. Different upper-case letters indicate significant differences by *t*-test (*p* < 0.01).

### 3.5. Effect of volatile compounds produced by JY-7-2L on hyphal growth of *S. rolfsii*

**FIGURE 5:**
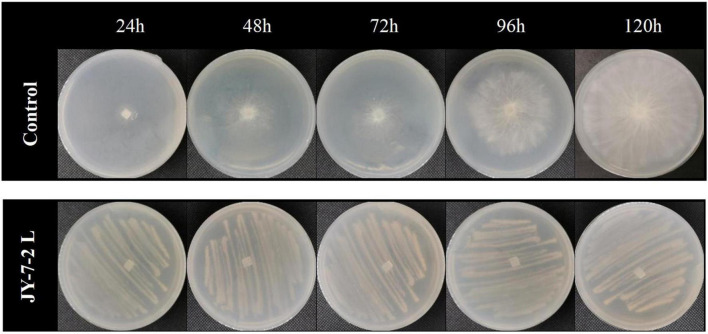
Effect of volatile compounds produced by JY-7-2L on hyphal growth of *Sclerotium rolfsii.*

Dual-culture assay by sealing two agar plates up and down was used to check the effect of volatile compounds in JY-7-2L on the hyphal growth of *S. rolfsii*. As shown in Table 4 and Figure 5, in the control treatment, the hyphal diameter of the pathogen increased over time and occupied the whole PDA plate after 96-h incubation, while pathogenic hyphal growth was entirely inhibited in the JY-7-2L co-culture treatment even after 120-h incubation. The results indicated that JY-7-2L was able to produce volatile compounds that completely inhibited the hyphal growth of *S. rolfsii*.

**TABLE 4:** Effect of volatile compounds produced by JY-7-2L on hyphal growth of *Sclerotium rolfsii*.

Strain	Incubation time (h)									
	**24 h**		**48 h**		**72 h**		**96 h**		**120 h**	
	**Diameter of hyphae (cm)**	**Inhibition rate (%)**	**Diameter of hyphae (cm)**	**Inhibition rate (%)**	**Diameter of hyphae (cm)**	**Inhibition rate (%)**	**Diameter of hyphae (cm)**	**Inhibition rate (%)**	**Diameter of hyphae (cm)**	**Inhibition rate (%)**
CK	1.53 ± 0.03 A	—	3.77 ± 0.03 A	—	5.57 ± 0.17 A	—	8.45 ± 0.19 A	—	8.50 ± 0.00 A	—
JY-7-2 L	0.00 ± 0.00 B	100.00	0.00 ± 0.00 B	100.00	0.00 ± 0.00 C	100.00	0.00 ± 0.00 C	100.00	0.00 ± 0.00 B	100.00

Data showed the means with a standard error of three replicates. Different upper-case letters indicated significant differences by *t*-test (*p* < 0.01).

### 3.6. Detection of secondary metabolites synthesize genes

**FIGURE 6:**
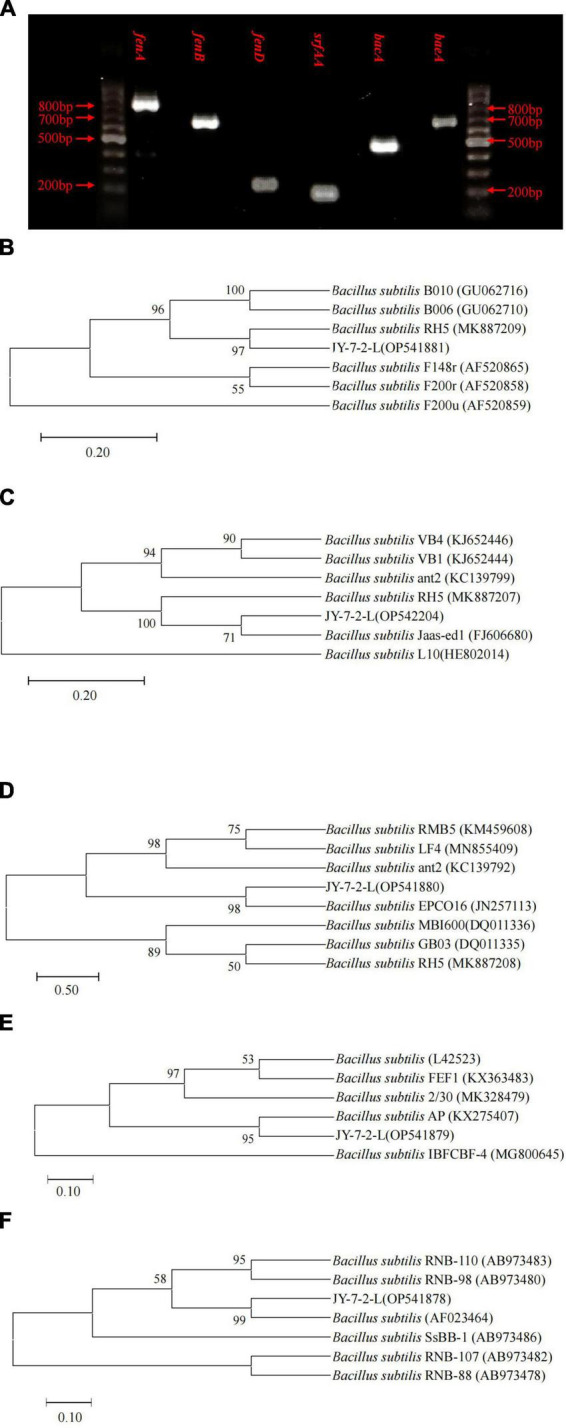
Detection of functional genes involved in antifungal metabolites synthesis of JY-7-2L. **(A)** Agarose gel-electrophoresis of PCR fragments of secondary metabolites synthesis genes of JY-7-2L. The Neighbor-Joining trees based on **(B)** surfactin (*srfAA*), **(C)** bacilysin (*bacA*), **(D)** *fenD*, **(E)** *fenB*, and **(F)** *fenA* of JY-7-2L and reference strains. Accession numbers were indicated in brackets. Bootstrap values greater than 50% were indicated on the branches.

We succeeded in amplifying four types of genes that are involved in antimicrobial lipopeptides, polyketides, and dipeptide synthesis. PCR amplification generated fragment products of 820, 670, 269, 200, 500, and 688 bp for *fenA, fenB*, *fenD*, *srfAA*, *bacA*, and *baeA*, respectively (Figure 6A). Phylogenetic trees based on *fenA, fenB*, *fenD*, *srfAA*, and *bacA* gene sequences all indicated that JY-7-2L belonged to *B. subtilis* (Figures 6B–F), which was consistent with the results of housekeeping genes.

### 3.7. Biocontrol efficacy of JY-7-2L on the southern blight of *A. carmichaelii* under field conditions

**FIGURE 7:**
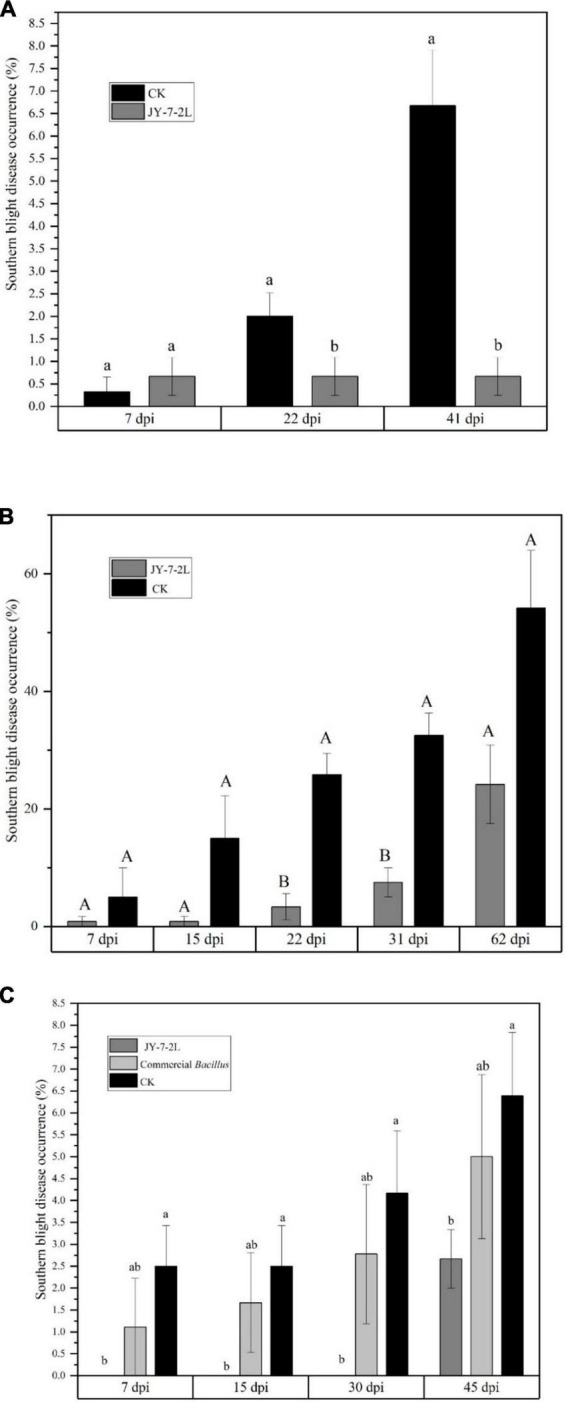
Biocontrol potential of JY-7-2L on southern blight of *Aconitum carmichaelii* by field experiments. **(A)** Biocontrol efficacy of JY-7-2L on southern blight of *A. carmichaelii* in the field site of Southwest University of Science and Technology (SWUST) and **(B)** in Qiangchuan field in 2021; **(C)** biocontrol efficacy of JY-7-2L on southern blight of *A. carmichaelii* in SWUST field in 2022. Different lower-case or capital letters indicated significant difference (*p* < 0.05 for lower-case letters and *p* < 0.01 for capital letters) according to one -way analysis of variance (ANOVA) with least significant difference (LSD) test.

Considering the great antagonistic activity of JY-7-2L *in vitro*, field experiments were conducted to evaluate the biocontrol efficacy of JY-7-2L on the southern blight of *A. carmichaelii*. The fermentation culture of JY-7-2L was applied to *A. carmichaelii* in the SWUST research field in May 2021. Ten milliliters of the fermentation culture were applied directly to the wounded parts of the roots of *A. carmichaelii* after removing the redundant lateral roots. The results showed that the southern blight disease occurrences from non-inoculation (CK) increased over time, while disease occurrences from JY-7-2L inoculation treatment were at a stable level (up to 41 days). Compared to CK, JY-7-2L significantly reduced the disease occurrences of southern blight in *A. carmichaelii* at 22 days post-inoculation (dpi) and this effective duration lasted until 41 dpi (Figure 7A).

To verify the biocontrol efficacy of JY-7-2L, field experiments were repeated in the Qingchuan area with a totally different soil type from that in Jiangyou, in August 2021. The field in Qingchuan has cultivated *A. carmichaelii* for over 10 years, and the southern blight severity was quite serious when we were conducting the experiment. Inoculation of fermentation culture was applied directly to the roots of *A. carmichaelii* since no removing lateral root management was needed in this area. As indicated in Figure 7B, the disease occurrences of non-inoculation treatment increased from 5 to 54% within 2 months. However, disease occurrences were prevented to a quite low level (0.8–7.5%) by JY-7-2L within 31 days (biocontrol efficacy = 76.9% at 31 dpi). The disease occurrence of JY-7-2L was 24%, which was 30% lower than CK (*p* = 0.073) at 62 dpi (biocontrol efficacy = 55.6%).

The field experiment was repeated in the same research field at SWUST in 2022. The management of *A. carmichaelii* during cultivation and inoculation of JY-7-2L was consistent with that in 2021. We used a commercial *B. subtilis* wettable powder product as a positive control. The results showed that disease occurrences caused by commercial *B. subtilis* products and non-inoculation treatments increased over time. Plant disease occurrence by commercial *B. subtilis* was lower than that by CK but with no significant difference (Figure 7C). However, no disease occurrence was detected by JY-7-2L treatment, even after 30 days of inoculation. At 45 dpi, the disease occurrence by JY-7-2L treatment was significantly lower than that by non-inoculation treatment (biocontrol efficacy = 58.26%) (Figure 7C). The biocontrol efficacy of commercial *B. subtilis* was 21.74% at 45 dpi, which was much lower than that of the JY-7-2L treatment (58.26%). Taken together, JY-7-2L showed a stronger biocontrol efficacy than a commercial *B. subtilis* product.

### 3.8. Plant growth promotion ability of JY-7-2L

**FIGURE 8:**
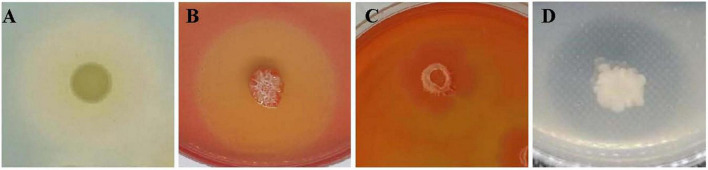
Plant growth promotion activity of JY-7-2L *in vitro*. **(A)** Siderophore halo on a chrome azurol-s (CAS) agar medium; **(B)** cellulose halo on a CMC agar medium; **(C)** glucanase halo on glucan agar medium; and **(D)** protease halo on skim milk agar medium.

**FIGURE 9:**
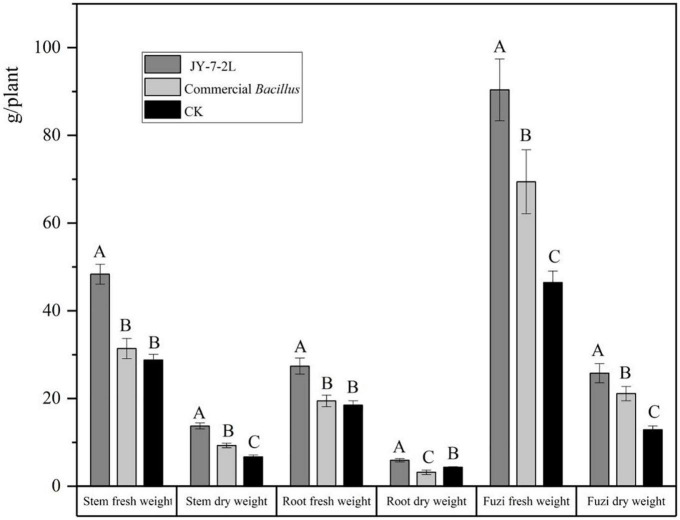
Effect of JY-7-2L and commercial *Bacillus subtilis* products on the fresh and dry weight of stem, main root, and lateral root (Fuzi) of *Aconitum carmichaelii*. The experiment was performed in the Southwest University of Science and Technology (SWUST) field in 2022. Different capital letters indicated significant differences (*p* < 0.01) based on one-way analysis of variance (ANOVA) with the least significant difference (LSD) test.

JY-7-2L was able to produce IAA, the amount of which was 3.4 mg/L (Supplementary Table 2). In addition, we also detected a positive halo for siderophore, cellulase, glucanase, and protease production after inoculation of JY-7-2L on the corresponding agar medium (Figure 8 and Supplementary Table 2). These results indicated that JY-7-2L showed potential for plant growth promotion. Therefore, the fermentation culture of JY-7-2L was applied to *A. carmichaelii* plants in a field experiment in the SWUST research field to confirm its plant growth promotion ability in 2022. As shown in Figure 9, the commercial *B. subtilis* product promoted fresh and dry weights of Fuzi compared to CK, while it had no effect on fresh and dry weights of stems and main roots. Conversely, inoculation of JY-7-2L increased significantly the fresh and dry weights of the stem, main root, and Fuzi of *A. carmichaelii*, respectively, compared to commercial *B. subtilis* products and non-inoculation treatments (*p* < 0.01). JY-7-2L inoculation treatment increased 105, 36.7, and 99.7% of the dry weight of the stem, main root, and Fuzi, respectively, relative to CK, while JY-7-2L promoted 48.0, 87.3, and 21.9% of the dry weight of the stem, main root, and Fuzi, respectively, compared to commercial *B. subtilis* product treatment. Therefore, JY-7-2L displayed substantial plant growth promotion potential under field conditions.

## 4. Discussion

Beneficial microbe-based biocontrol has attracted great attention in recent years for the control of plant diseases because of its minimal impact on the environment and food safety. The most commonly used biocontrol agents for southern blight disease caused by *S. rolfsii* were *Trichoderma* spp. and *Pseudomonas* spp., for example, in peanut, soybean, tomato, and sugar beet (Li et al., 2019; Ram et al., 2020). This study aimed to investigate the biocontrol and plant growth promotion efficacy of an endophytic bacteria, JY-7-2L, isolated from the leaves of *A. carmichaelii* on southern blight *in vitro* and under field conditions.

JY-7-2L was identified as *B. subtilis* based on morphological and molecular characterization of multi-locus housekeeping genes (16S, *atpD*, *gyrA*, and *rpoB*). *B. subtilis* has been widely demonstrated to prevent many pathogens under laboratory conditions. For example, *B. subtilis* IBFCBF-4 has shown great biocontrol potential against the *Fusarium* wilt of watermelon (Zhu et al., 2020). *B. subtilis* Tpb55 exhibited strong antagonism against *Phytophthora nicotianae*, which causes black shank in tobacco (Han et al., 2015). Consistently, in the current study, *B. subtilis* JY-7-2L showed strong antagonistic activity against *S. rolfsii* and *F. oxysporum in vitro* and on *A. carmichaelii* root slices (Table 1 and Figure 2). We further found that a cell-free culture of JY-7-2L significantly inhibited the hyphal growth, sclerotia formation, and germination of *S. rolfsii*. Sclerotia is a special structure of *S. rolfsii* and could survive for several years in the soil even under harsh conditions, which made it difficult to be controlled by rotational management or chemical fungicides (Li et al., 2019; Ram et al., 2020). Sclerotia are generated from vegetative hyphae but germinate once conditions are favorable and develop into hyphae, which then infect the plants (Chen et al., 2020). Therefore, one of the main roles of the biocontrol agents of *S. rolfsii* is to inhibit sclerotia germination. Li et al. (2019) reported that *P. griseofulvum* CF3 culture filtrate inhibited *S. rolfsii* sclerotia germination, whose inhibition rate was 44.2% after 48 h of incubation, but the inhibition rate was reduced to 8.3% at 96 h. *B. velezensis* LHSB1 culture filtrate showed effective suppression capacity for *S. rolfsii* sclerotia germination, with inhibition rates ranging from 96.7 to 98.4% within 72 h of incubation (Chen et al., 2020). In our study, *B. subtilis* JY-7-2L cell-free culture filtrate showed a 100% inhibition rate to *S. rolfsii* sclerotia germination even after 120 h of incubation (Table 3 and Figure 4). These results indicated that JY-7-2L may produce secondary metabolites, which played a pivotal role in suppressing the hyphal growth, sclerotia formation, and germination of *S. rolfsii*. For direct antagonistic targets in the biocontrol of pathogens, the common direct-interacting part with pathogenic fungal strains is the cell wall and its components. In this respect, hydrolytic enzymes are good tools used by biocontrol agents such as chitinase, glucanases, cellulases, and proteases (Dimkić et al., 2022). These enzymes are capable of breaking down glycosidic and peptide bonds that join the constituting polysaccharides and proteins in a synergistic way and promote the lysis of fungal cell walls (Jadhav et al., 2017; Dimkić et al., 2022). We confirmed that JY-7-2L was capable of producing cellulase, glucanase, and protease (Figures 8B–D). This may be one of the antagonistic mechanisms used by JY-7-2L. Another interesting group of secondary metabolites involved in direct pathogen suppression are antimicrobial metabolites such as bacteriocins, peptides and lipopeptides, polyketides, and siderophores (Fira et al., 2018; Gu et al., 2020). Noteworthy, siderophore also showed plant growth promoting properties (Kloepper et al., 1980). We demonstrated the production of siderophore by JY-7-2L on CAS medium (Figure 8A). By PCR amplification assay, we succeeded in amplifying six genes, which are involved in the synthesis of Fengycin, surfactin, bacilysin, and bacillaene, respectively, from the genomic DNA of JY-7-2L. Fengycin is a cyclic lipopeptide with strong antifungal, antibacterial, and insecticidal activity (Maksimov et al., 2020; Dimkić et al., 2022). Surfactin, a lipopeptide, plays pivotal roles in swarming motility, biofilm formation, plant tissue colonization, and competition for nutrients and space, and retains some synergistic effect on the antifungal activity of iturin A (Ben Khedher et al., 2021; Dimkić et al., 2022). Bacilysin is a dipeptide with antifungal activity (Wang et al., 2016). Bacillaene is a polyketide showing antifungal activity (Um et al., 2013; Dimkić et al., 2022). Therefore, the production of lipopeptides, dipeptides, polyketides, and siderophores may be another mode of action to inhibit *S. rolfsii* by JY-7-2L. In addition, *Bacillus* strains have been proven to produce various volatile compounds (such as aldehyde, ketone, alcohol, acid, pyrazine, and ester), which displayed an inhibitory effect on certain pathogens (Dimkić et al., 2022). For example, dimethyl disulfide (DMDS) produced by the *B. cereus* C1L strain was able to directly suppress mycelial growth and conidial germination of *Cochliobolus heterostrophus* (Huang et al., 2012). Zhang et al. (2020) showed that volatile compounds produced by *B. subtilis* strain ZD01 reduced the colony size and mycelial penetration and caused serious morphological changes in *Alternaria solani*, which causes early blight disease in potatoes. The mycelial growth of the pathogenic fungal strain *Alternaria iridiaustralis* was inhibited by volatile organic compounds produced by *B. velezensis* L1 (1 × 10^9^ CFU/mL) by approximately 92.86% (Ling et al., 2022). Consistent with these studies, we found that volatile compounds produced by JY-7-2L completely and directly repressed the hyphal growth of *S. rolfsii* (Table 4 and Figure 5). Identifying antagonistic lipopeptides, polyketides, and volatile compounds produced by JY-7-2L would be very helpful for elucidating the antagonistic mechanism of JY-7-2L and mining potential biocontrol products. Taken together, JY-7-2L (cells, cell-free culture filtrate, and/or volatile compounds) could significantly inhibit hyphal growth, sclerotia formation, and germination of *S. rolfsii*, which may effectively decrease the infection rate of *S. rolfsii* on *A. carmichaelii* plants, indicating the great biocontrol potential of JY-7-2L on southern blight.

Considering the promising antagonistic activity of JY-7-2L, we further evaluated the biocontrol efficacy of JY-7-2L on the southern blight of *A. carmichaelii* in a series of field experiments. Adaption to different soil conditions is very important for the effectiveness and persistence of biocontrol agents (Mazzola and Freilich, 2017; De Silva et al., 2019; Niu et al., 2020). Previously, Li et al. (2019, 2020) reported that powdered *P. griseofulvum* CF3 and two *Streptomyces* strains reduced around 20% of southern blight disease occurrences, respectively, in *A. carmichaelii* in field experiments in Shaanxi Province. In our study, two fields with distinguished environmental conditions were selected to investigate the biocontrol efficacy of JY-7-2L, i.e., the geo-authentic area in Jiangyou (SWUST research field, river plain, paddy soil, 500 m a.s.l., and mountainous cultivation area in Qingchuan (dark brown forest soil, 1,300 m a.s.l.), which provided mother roots of *A. carmichaelii* for cultivation in Jiangyou area. The soil in the SWUST research field was the first year to plant *A. carmichaelii*, so the natural southern blight disease occurrence was low. Nevertheless, we still found that JY-7-2L fermentation culture application to the roots significantly reduced disease occurrence compared to non-inoculation treatment in a 2-year continuous experiment (Figures 7A, C). In this study, *A. carmichaelii* has been cultivated in the Qingchuan area for more than 10 years. Before we started the experiment in Qingchuan, some of the *A. carmichaelii* plants in the field were already infected by *S. rolfsii* and exhibited wilt symptoms, with white hyphae and brown sclerotia of *S. rolfsii* appearing on the soil surface. Therefore, the southern blight disease occurrence in Qingchuan fields was generally higher (up to 60%). We found that the JY-7-2L fermentation culture reduced 30% (*P* < 0.05) of the disease occurrence compared to CK (Figure 7B). In addition, all the field experiments showed that JY-7-2L displayed a persistent biocontrol capacity (45 days in SWUST and 62 days in Qingchuan). These results indicated that JY-7-2L showed good adaptation ability to different soil conditions with a long effective duration. JY-7-2L also showed better biocontrol efficacy compared to commercial *B. subtilis* products (Figure 7C). With the exception of seed-borne endophytes, the majority of endophytes originate from soil or air commonly through natural openings or wounds of the host plants (Eljounaidi et al., 2016). Endophytes are usually host-specific (Tewari et al., 2019), and they are likely to interact more closely with the host plants with a good colonizing ability compared to soil or rhizospheric microbes (Rosenblueth and Martínez-Romero, 2006; Eljounaidi et al., 2016; Santoyo et al., 2016). Therefore, we speculated that JY-7-2L, being a bacterial endophyte of the *A. carmichaelii* plant, may have better colonization ability on its original host than commercial *B. subtilis*. Further experiments are needed to compare the colonization abilities of JY-7-2L and commercial *B. subtilis*.

Furthermore, JY-7-2L also significantly increased fresh and dry weights of the stem, main root, and lateral roots of *A. carmichaelii* compared to CK and commercial *B. subtilis* product treatments (Figure 9), which showed good plant growth promotion potential. Mechanisms for plant growth promotion by bacterial endophytes involve facilitating the acquisition of resources from the environment, including nitrogen, phosphorous, and iron providing plant hormones such as auxin, cytokinin, or ethylene, or adjusting microbial communities in the rhizosphere, and reducing plant diseases (Santoyo et al., 2016). JY-7-2L was able to produce IAA and siderophore, and inhibit pathogenic fungal growth, which may be the reasons that promote plant growth (Figure 8 and Supplementary Table 2).

## 5. Conclusion

Collectively, this study identified an endophytic bacterium *B. subtilis* JY-7-2L, which was isolated from *A. carmichaelii* plants and investigated its biocontrol and plant growth promotion potential. JY-7-2L showed strong antagonistic activity against the southern blight pathogen, i.e., *S. rolfsii in vitro* and on *A. carmichaelii* root slices. The possible mechanisms involved the production of hydrolytic enzymes including glucanase, cellulase, and protease, and the production of antimicrobial compounds such as lipopeptide, polyketides, and volatile compounds. Two-year field experiments in two different ecological regions showed that JY-7-2L could significantly reduce southern blight disease occurrences compared to CK or a commercial *B. subtilis* product with persistent activity. In addition, JY-7-2L showed great plant growth promotion ability *in vitro* and significantly promoted plant biomass under field conditions. Taken together, *B. subtilis* JY-7-2L represented a highly effective biocontrol agent against the southern blight of *A. carmichaelii*.

## Data availability statement

The datasets presented in this study can be found in online repositories. The names of the repository/repositories and accession number(s) can be found in the article/Supplementary material.

## Author contributions

LZ designed the experiments, analyzed data, and prepared the manuscript. QW, RW, YZ, QSW, ML, KY, and WD performed the experiments. JH revised the manuscript. All authors reviewed the manuscript and approved the final version.

## Footnotes

1
http://www.gov.cn/zhengce/zhengceku/2022-03/15/content_5679257.htm
