# Supplementary material for: Biocontrol and plant growth promotion potential of endophytic Bacillus subtilis JY-7-2L on Aconitum carmichaelii Debx
Source: Front Microbiol. 2023 Jan 10;13:1059549. doi: 10.3389/fmicb.2022.1059549 (PMC9871935; doi:10.3389/fmicb.2022.1059549)
Supplement: Supplementary file 1 [file Data_Sheet_1.docx]

**Supplementary TABLE S1** Primers and PCR procedures used for housekeeping genes amplification of JY-7-2L

| Gene | Primers | Reaction procedure |
| --- | --- | --- |
| 16S rRNA | 27F: 5'-AGAGTTTGATCCTGGCTCAG-3'; | 94℃ for 3 min, 30 cycles of 94℃ for 1 min, 56℃ for 1 min, 72℃ for 2 min, final extension for 72℃ 10 min |
|  | 1492R: 5'-TACGGCTACCTTGTTACGACTT-3' |  |
| *atpD* | atpD-0F: 5'-CGGCATCAAGGTTATCGACCTG-3'; | 94℃ for 3 min, 35 cycles of 94℃ for 15 s, 55℃ for 30 s, 72℃ for 30 s, final extension for 72℃ 10 min |
|  | atpD-7R: 5'-ACCAGCGGATCCAGCTGACG-3' |  |
| *gyrA* | gyrA2F: 5'-ATGAGCGATCTGGCCAGAGA-3'; | 98℃ for 2 min, 35 cycles of 98℃ for 30 s, 57℃ for 1 min, 72℃ for 45 s, final extension for 72℃ 10 min |
|  | gyrA9R: 5'-CGCGCCTTGTTCACCTGATA-3' |  |
| *rpoB* | rpoB-f: 5'-AGGTCAACTAGTTCAGTATGGAC-3'; | 94℃ for 3 min, 30 cycles of 94℃ for 30 s, 54℃ for 45 s, 72℃ for 30 min, final extension for 72℃ 10 min |
|  | rpoB-r: 5'-AAGAACCGTAACCGGCAACTT-3' |  |

**Supplementary TABLE S2** Characterization of growth promotion activity of JY-7-2L

| Isolate | IAA  (mg/L) | Siderophore (D/d) | Cellulase  (D/d) | Glucanase  (D/d) | Protease  (D/d) |
| --- | --- | --- | --- | --- | --- |
| JY-7-2L | 3.40±0.22 | 3.50±0.05 | 3.96±0.04 | 1.61±0.04 | 2.82±0.09 |

Data showed the means with standard error of three replicates. D, referred to diameter of halo; d, referred to diameter of the bacterial colony.

*Klebsiella michiganensis* THO-011 (AP022547)

*Klebsiella pasteurii* SB6412 (CABGHC010000005)

*Enterobacter asburiae* 1808-013 (AP019632)

*Pantoea vagans* LMG 24199 ^T^ (CP038853)

*Serratia grimesii* BXF1 (LT883155)

*Serratia liquefaciens* S1 (CP048784)

*Pseudomonas monsensis* PGSB 8459 ^T^ (CP077087)

*Pseudomonas putida* NBRC 14164 ^T^ (AP013070)

*Microbacterium wangchenii* dk512 ^T^ (CP038266)

*Bacillus subtilis* subsp. *subtilis* str. 168 (AL009126)

**JY-7-2L(OP391485)**

*Mesobacillus zeae* JJ-247^T^ (QWVT01000038)

*Microaerobacter geothermalis* DSM 22679 ^T^ (JAKIHL010000005)

*Paraliobacillus salinarum* G6-18 ^T^ (JACEFA010000002)

*Alkalibacillus aidingensis* YIM 98829 ^T^ (JABXWU010000007)

*Lysinibacillus antri* SYSU K30002 ^T^ (RYYR01000010)

*Anoxybacillus salavatliensis* DSM 22626 ^T^ (JANGZY010000001)

*Priestia taiwanensis* DSM 27845 ^T^ (JAFBEL010000006)

*Pseudomonas coronafaciens* pv. *oryzae* str. 16 (CP046035)

*Serratia ureilytica* T6 (CP071320)

0.20

**Supplementary FIGURE S1**. Phylogenetic tree based on *rpoB* gene generated by a neighbor-joining method. Bootstrap value was 1,000 and those greater than 50% were indicated. Bars=0.21 nucleotide substitution per site.

*Paraliobacillus salinarum* G6-18 ^T^ (JACEFA010000002)

*Priestia taiwanensis* DSM 27845 ^T^ (JAFBEL010000006)

*Anoxybacillus salavatliensis* DSM 22626 ^T^ (JANGZY010000001)

*Lysinibacillus antri* SYSU K30002 ^T^ (RYYR01000010)

*Alkalibacillus aidingensis* YIM 98829 ^T^ (JABXWU010000007)

*Bacillus subtilis* NCTC4744 (UATI01000005)

**JY-7-2L (OP391483)**

*Microaerobacter geothermalis* DSM 22679 ^T^ (JAKIHL010000005)

*Mesobacillus zeae* JJ-247 ^T^ (QWVT01000038)

*Pseudomonas koreensis* JCM 14769 ^T^ (BMOG01000001)

*Pantoea alhagi* NX-11 (CP097983)

*Enterobacter asburiae* 1808-013 (AP019632)

*Klebsiella michiganensis* THO-011 (AP022547)

*Pantoea ananatis* PA13 (CP003085)

*Pantoea eucalypti* LMG 24197 ^T^ (CP045720)

*Bacillus subtilis* subsp. *subtilis* str. 168 (AL009126)

*Bacillus subtilis* NCIB 3610 (CP020102)

*Bacillus subtilis* LBUM979 (CP065789)

*Pseudomonas putida* NBRC 14164 ^T^ (AP013070)

0.20

**Supplementary FIGURE S2**. Phylogenetic tree based on *atpD* gene generated by a neighbor-joining method. Bootstrap value was 1,000 and those greater than 50% were indicated. Bars=0.21 nucleotide substitution per site.

*Bacillus subtilis* subsp. *subtilis* VCRCB471(EF687841)

*Bacillus subtilis* MMA7 (JX977130)

*Bacillus subtilis* 230-27 (JX977131)

*Bacillus subtilis* NRRL NRS-744 ^T^ (EU138658)

*Bacillus subtilis* subsp. *spizizenii* ATCC 6633 (JX861882)

**JY-7-2L (OP391484)**

*Bacillus subtilis* CC15 (JX977124)

*Bacillus vallismortis* NRRL B-14890 ^T^ (EU138601)

*Bacillus subtilis* BLB277 (KU847916)

*Bacillus rugosus* SPB7 ^T^ (MK473855)

*Bacillus inaquosus* NRRL B-23058 (EU138609)

*Bacillus inaquosus* NRRL BD-571 (EU138633)

*Bacillus inaquosus* NRRL B-23052 (EU138605)

*Mesobacillus zeae* JJ-247^T^ (QWVT01000038)

*Alkalibacillus aidingensis* YIM 98829 ^T^ (JABXWU010000007)

*Microaerobacter geothermalis* DSM 22679 ^T^ (JAKIHL010000005)

*Anoxybacillus salavatliensis* DSM 22626 ^T^ (JANGZY010000001)

*Priestia taiwanensis* DSM 27845 ^T^ (JAFBEL010000006)

*Paraliobacillus salinarum* G6-18 ^T^ (JACEFA010000002)

*Lysinibacillus antri* SYSU K30002 ^T^ (RYYR01000010)

0.050

**Supplementary FIGURE S3** Phylogenetic tree based on *gyrA* gene generated by a neighbor-joining method. Bootstrap value was 1,000 and those greater than 50% were indicated. Bars=0.01 nucleotide substitution per site.
